# Screening, Identification, and Characterization of Two Folate-Producing *Lactiplantibacillus plantarum* Strains

**DOI:** 10.3390/foods15101705

**Published:** 2026-05-13

**Authors:** Bo Pang, Haobin Mo, Wenxin Zhang, Wenqiong Wang, Dawei Chen, Ruixia Gu, Yujun Huang

**Affiliations:** 1 School of Food Science and Engineering, Yangzhou University, Yangzhou 225127, China; 2 Key Laboratory of Probiotics and Deep Processing of Dairy Products, Yangzhou University, Yangzhou 225127, China

**Keywords:** folate, synthesis pathway, genetic transcription, lactic acid bacteria, *Lactiplantibacillus plantarum*, probiotic

## Abstract

In this study, two folate-producing *Lactiplantibacillus plantarum* bacteria, known as Grx1201 and Grx1202, were screened with folic acid assay medium (FAAM). When 4 μg/100 mL of exogenous folic acid was added to FAAM, the maximum folate production of Grx1201 and Grx1202 reached 9.01 μg/100 mL and 10.47 μg/100 mL, respectively, which were 2.30 and 2.14 times greater than those of the control. High survival rates of Grx1201 and Grx1202 were observed at pH 3.0. Antibiotic resistance, biogenic amine-synthesizing ability, and hemolytic ability analyses revealed that Grx1201 and Grx1202 were safe for biological application. To determine the reason for the synthesis of folate, the transcription levels of key genes in the folate synthesis pathway in Grx1201 and Grx1202 were analyzed and compared. Grx1201 and Grx1202 reported here can greatly improve the folate content in functional foods.

## 1. Introduction

Folate is a conjugated complex of *p*-aminobenzoic acid (*p*ABA), pteridine, and _L_-glutamic acid linked by covalent bonds [1]. It stimulates the immune function of white blood cells to improve resistance to external viruses and bacteria and promotes the proliferation of red blood cells to prevent anemia [2,3]. In addition, folate is involved in the synthesis and regulation of neurotransmitters, which help maintain cognitive ability [4,5]. The Nordic Nutrition recommends a daily folate intake of 300 μg for men, 400 μg for women, and 500 μg for pregnant women [6]. Folate can be metabolized into 5-methyltetrahydrofolate, which is a key methyl donor in the process of homocysteine methylation [7]. Folate deficiency prevents the efficient conversion of homocysteine to methionine, leading to the accumulation of homocysteine [8]. The increased homocysteine not only promotes thrombosis by damaging vascular endothelial cells to induce cardiovascular disease [9], but also affects placental vascularization and embryo implantation to elevate the risk of miscarriage [10]. Furthermore, it interferes with the closure of the neural tube, resulting in the occurrence of neural tube defects [11]. Because natural folate in foods is easily degraded, many individuals cannot obtain sufficient folate through diet alone [12]. Therefore, folate deficiency remains a common concern.

Folate-fortified foods and folate medications are used to replenish folate levels for maintaining health. However, these products are often produced by chemical synthesis, which may increase associated health risks and environmental burdens [13,14]. For example, long-term excessive intake of synthetic folate may mask the symptoms of vitamin B12 deficiency, alter the activity of hepatic dihydrofolate reductase, and promote cancer development [15,16,17,18]. Compared with synthetic folate, natural folate has greater safety [19].

Folate-producing microorganisms are widely used to obtain natural folate [20,21]. Certain microbes, including lactic acid bacteria, can synthesize folate [22,23]. Microbially produced folate is more readily absorbed and may more effectively meet physiological requirements [24,25]. *Lactiplantibacillus plantarum*, a lactic acid bacterium, plays important roles in immunological regulation, inhibits the proliferation of pathogenic bacteria, decreases serum cholesterol levels, maintains intestinal flora balance, promotes nutrient absorption, relieves lactose intolerance, and prevents the reproduction of tumor cells [26,27,28,29]. *L. plantarum* is extremely well adapted to different niches due to a highly flexible genome with life-style islands mainly related to the utilization of carbohydrates [30]. Most members of the *Lactiplantibacillus* are normal representatives of the human intestinal microbiota and are generally recognized as safe (GRAS) [31]. It was shown that the vitamin-producing strain *L. plantarum* GSLP-7 V could increase the level of serum folate in model rats with folic acid deficiency [32]. Identification of *L. plantarum* strains with folate-producing ability has triggered the growing interest [33].

In this study, we screened two folate-producing *L. plantarum* strains, Grx1201 and Grx1202. To improve the potential for application in functional foods, folate production by Grx1201 and Grx1202 was enhanced. The resistance of Grx1201 and Grx1202 to acid and bile salt was characterized. The biosafety of Grx1201 and Grx1202 including antibiotic resistance, biogenic amine-synthesizing ability, and hemolytic ability was evaluated. To explore the basis of folate synthesis, the transcription levels of key genes in the folate biosynthesis pathway were analyzed and compared between Grx1201 and Grx1202. This work enriches the library of folate-producing *L. plantarum* strains.

## 2. Materials and Methods

### 2.1. Microorganisms and Reagents

The strains used in this study are listed in Supplementary Table S1 and were preserved in our laboratory. The *L. rhamnosus* GG and *Staphylococcus aureus* ATCC 6538 strains were obtained from the China Center for Type Culture Collection (Wuhan, China). Folic acid assay medium (FAAM) was purchased from HOPEBIO (Qingdao, China). Columbia agar with sheep blood mixture was purchased from OXOID (Basingstoke, UK). PrimeSTAR HS DNA Polymerase was purchased from TaKaRa (Dalian, China). The bacteria genome extraction kit, RNA extraction kit, and HiScript II 1st Strand cDNA Synthesis Kit (+gDNA wiper) were purchased from Vazyme Biotech (Nanjing, China). The primers used for amplifying 16S rDNA were synthesized by GENEWIZ (Suzhou, China). The remaining analytical-grade reagents were purchased from Sigma-Aldrich (Shanghai, China).

### 2.2. Inoculum Preparation

The preserved strains were inoculated on de Man, Rogosa, and Sharpe (MRS) plates and grown at 37 °C for 48 h. A single colony was collected in a 50 mL test tube containing 10 mL of MRS medium and cultivated at 37 °C for 24 h. The fermentation broth was centrifuged at 4 °C and 8000 rpm for 10 min via Sorvall Legend 21 R (Thermo Fisher Scientific Co., Ltd., Shanghai, China), after which the supernatant was removed. Strains at the bottom of the tube were washed with 0.85% (*w*/*v*) NaCl solution three times and resuspended as inocula.

### 2.3. Cultivation of Strains in FAAM

The inocula were transferred into 50 mL test tubes containing 10 mL FAAM with an inoculum size of 2% (*v*/*v*) and then cultivated at 37 °C for 16 h. Then, the fermentation broth was centrifuged at 4 °C and 8000 rpm for 10 min to separate the isolates from the supernatant. The isolates were washed with 0.85% (*w*/*v*) NaCl solution three times and resuspended. The optical density at 580 nm (OD_580_) of the isolates and the folate content in the supernatant were analyzed.

### 2.4. Cultivation of Strains in FAAM Supplemented with Different Concentrations of Folate

The inocula were transferred into a 50 mL test tube containing 10 mL of FAAM supplemented with 2 μg/100 mL, 4 μg/100 mL, 6 μg/100 mL, 8 μg/100 mL, or 10 μg/100 mL of folate with an inoculum size of 2% (*v*/*v*) and then cultivated at 37 °C for 16 h. The isolates and supernatants were collected and disposed as described above. The OD_580_ values of the isolates and the folate content in the supernatant were determined. The folate yield was equal to the folate content in the supernatant minus the amount of folate added to FAAM.

### 2.5. Analysis of the Folate Content

We used 96-well sterile microplates for the experiment. The folate concentration was quantified by conducting a previously reported microbiological assay on the basis of the growth of the indicator bacterium *L. rhamnosus* ATCC 7469 with 5-formyltetrahydrofolate as the calibrant [34]. Two dilutions were made from the supernatant using 0.5% (*w*/*v*) sodium ascorbate solution and ten levels of calibrant (0–12 μg/100 mL) in each plate. The plates were cultured at 37 °C for 48 h and turbidity was determined at 595 nm via a Multiskan Go microplate reader (Thermo Fisher Scientific Co., Ltd., Shanghai, China). The folate content was calculated by comparing the turbidity of the culture broth with that of the standard curve generated from calibrant. The recovery rate, relative standard deviation, and detection limit of the folate quantification method were 95 to 105%, <15%, and 0.3 μg/100 mL.

### 2.6. Species Identification

Selected strains were identified via an API 50 CHL kit (bioMérieux SA, Marcy l’Etoile, France) based on the carbohydrate fermentation patterns. The genomes of the selected strains were obtained via a bacterial genome extraction kit. The specific primers 16-F and 16-R were used to amplify the 16S rDNA sequences of selected strains via the extracted genomes as templates (Supplementary Table S2). The amplified 16S rDNAs were analyzed via agarose gel nucleic acid electrophoresis, sequenced via GENEWIZ (Suzhou, China), and blasted via NCBI BLAST (2.16.0) [35].

### 2.7. Analysis of Acid and Bile Salt Resistance

First, 1 mL of inoculum was inoculated in 9 mL of artificially simulated gastric juice, which consisted of 5 × 10^5^ μg/100 mL NaCl and 3 × 10^5^ μg/100 mL pepsin, with pH values of 2.0 and 3.0, and then cultured at 37 °C. The number of viable bacteria at 0 h and 3 h was determined via the plate counting method. The survival rate was defined as the ratio of the number of viable bacteria at 3 h to the number of viable bacteria at 0 h multiplied by 100%.

Then, 1 mL of inoculum was inoculated in 9 mL of MRS medium supplemented with 0.1 (*w*/*v*) or 0.3 (*w*/*v*) bile salt and cultured at 37 °C. The number of viable bacteria at 0 h and 3 h was determined. The survival rate was calculated as described above.

### 2.8. Biosafety Evaluation

#### 2.8.1. Antibiotic Resistance Analysis

The sensitivity of folate-producing strains to various antibiotics was evaluated via the Kirby–Bauer method [36]. The antibiotic contents of the drug-sensitive tablets and resistance assessment criteria are listed in Supplementary Table S3. First, the OD_580_ value of each isolate was adjusted to 0.6 with phosphate-buffered saline. Then, 100 μL of each isolate was sampled and coated on the surface of an MRS plate. When the plate dried, the drug-sensitive tablet was attached to its surface. After the plate was left undisturbed for 30 min, it was cultured at 37 °C for 24 h, after which the diameter of the inhibition zone was measured. The food-grade strain *L. rhamnosus* GG was used as a control.

#### 2.8.2. Analysis of the Biogenic Amine Synthesizing Ability

To detect the biogenic amine-synthesizing ability, the inocula were inoculated on a biogenic amine test medium containing 10^6^ μg/100 mL lysine, 10^6^ μg/100 mL tyrosine, 10^6^ μg/100 mL ornithine, or 10^6^ μg/100 mL histidine and then cultured at 37 °C for 48 h. The color of the medium was observed. The *S. aureus* ATCC 6538 strain was used as a control. The components of the test medium are listed in Supplementary Table S4.

#### 2.8.3. Hemolytic Ability Analysis

To assess the hemolytic ability, the inocula were inoculated on Columbia agar with sheep blood mixture [37] and then cultured at 37 °C for 48 h. The appearance of a transparent circle was recorded. The *S. aureus* ATCC 6538 strain was used as a control.

### 2.9. Determination and Transcription Level Analysis of Key Genes in the Folate Synthesis Pathway

The genomes of the folate-producing strains were obtained using a bacterial genome extraction kit. The specific primers listed in Supplementary Table S2 were used to amplify key genes related to folate synthesis, including *fol*E, *fol*Q, *fol*B, *fol*K, *fol*P, *fol*C_1_, *fol*A, and *fol*C_2_, with the extracted genomes used as templates. The amplified gene fragments were analyzed via agarose gel nucleic acid electrophoresis.

To analyze the transcription levels of key genes, quantitative real-time polymerase chain reaction (qRT-PCR) was performed. Isolates were collected after 16 h of growth in FAAM supplemented with 4 μg/100 mL folate. Total RNA was extracted via a bacterial RNA extraction kit. The purified RNA was reverse-transcribed to cDNA via a HiScript II 1st Strand cDNA Synthesis Kit (+gDNA wiper) with the specific primers listed in Supplementary Table S2. Next, qRT-PCR was performed via qTOWER3G (Analytik Jena AG, Jena, Germany). The fold changes in key genes in different isolates were quantified via the 2^–^^∆∆Ct^ method [38]. The 16S rDNA was used as the internal reference gene.

### 2.10. Statistical Analysis

Statistical analysis was performed via SPSS Statistics 29.0. Data of three independent experiments were statistically analyzed using one-way analysis of variance (ANOVA) followed by a Tukey’s post-hoc test. The results were expressed as mean ± standard deviation. The differences among the samples were considered statistically significant at *p* < 0.05.

## 3. Results and Discussion

### 3.1. Screening of Folate-Producing Strains

**Figure 1:**
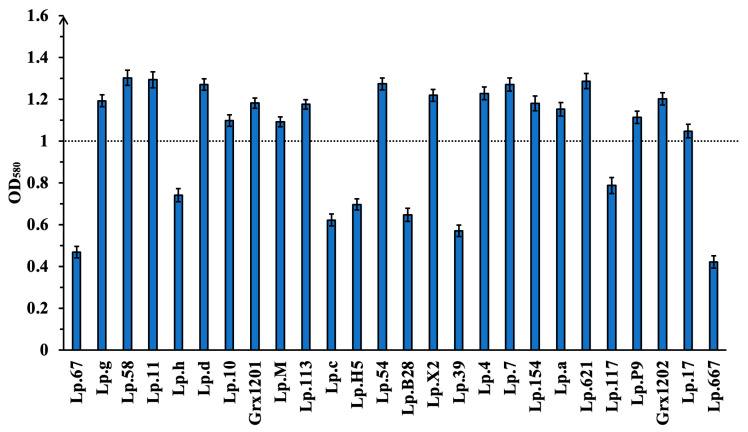
Growth of 26 strains isolated from human feces, pickle, chimichurri sauce, marinated cucumber, and milk fan after 16 h in FAAM, quantified by OD_580_ values.

FAAM is commonly used for screening folate-producing microorganisms. Mahara et al. (2021) reported that strains grown in FAAM with OD_580_ values > 1.0 after incubation for 16 h could produce folate [39]. Based on this criterion, 26 strains isolated from human feces, pickle, chimichurri sauce, marinated cucumber, and milk fan were inoculated into FAAM, and the OD_580_ values were determined after 16 h. As shown in Figure 1, the growth of these strains differed significantly, and 18 isolates exhibited OD_580_ values > 1.0, indicating that these strains could utilize the nutrients in FAAM for folate synthesis. Therefore, the extracellular folate yields of these isolates were further evaluated. Six isolates produced detectable folate (Table 1), among which strains Grx1201, Grx1202, Lp.M, and Lp.17 yielded more than 3.0 μg/100 mL of folate and were selected for subsequent experiments.

Mahara et al. (2021) screened a folate-producing strain, *L. fermentum* JK13, from kefir granules with an extracellular folate yield of 2.427 μg/100 mL in FAAM [39]. Purwandhani et al. (2018) isolated 16 *L. plantarum* strains from traditional fermented milk (dadih), with folate production ranging from 1.243 to 2.784 μg/100 mL in skim milk medium, and the folate yield of *L. plantarum* Dad-13 as the control reached 2.927 μg/100 mL [6]. Greppi et al. (2017) reported that *L. fermentum* 8.2 produced 2.9 μg/100 mL of folate in FAAM [40]. Mosso et al. (2018) isolated *L. sakei* T3MS2, *L. fermentum* T3M3, *Lactiplantibacillus* sp. T3Y6, and *L. casei* 3T3M3 from the traditional Andean fermented potato product tocosh, with extracellular folate yields of no more than 3.0 μg/100 mL in FAAM [41]. Abubakr et al. (2025) isolated three *Enterococcus faecium* bacteria from yogurt and milk samples, with the folate production levels of 0.226 to 0.830 μg/100 mL [42]. Compared with these strains, Grx1201, Grx1202, Lp.M, and Lp.17 showed higher extracellular folate yields in FAAM.

### 3.2. Effect of Different Concentrations of Folate on the Growth and Folate Synthesis of Screened Strains

**Figure 2:**
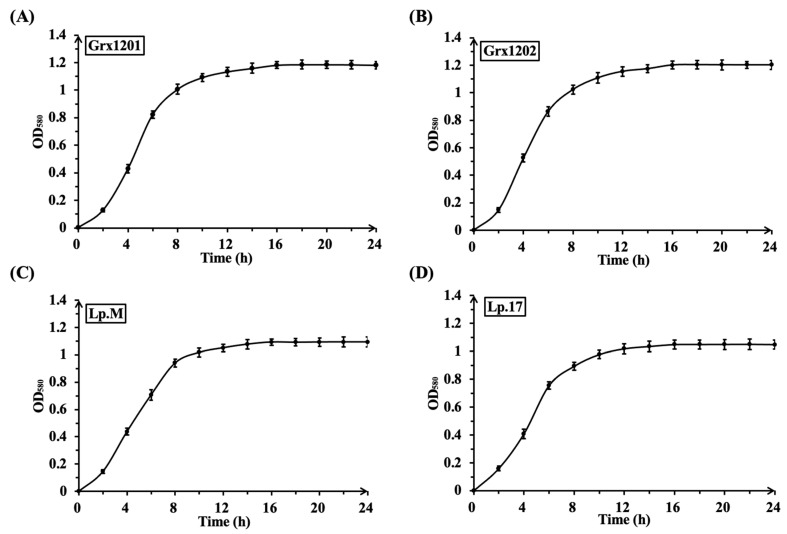
The growth curves of screened strains in FAAM: (**A**) Grx1201, (**B**) Grx1202, (**C**) Lp.M, and (**D**) Lp.17.

**Figure 3:**
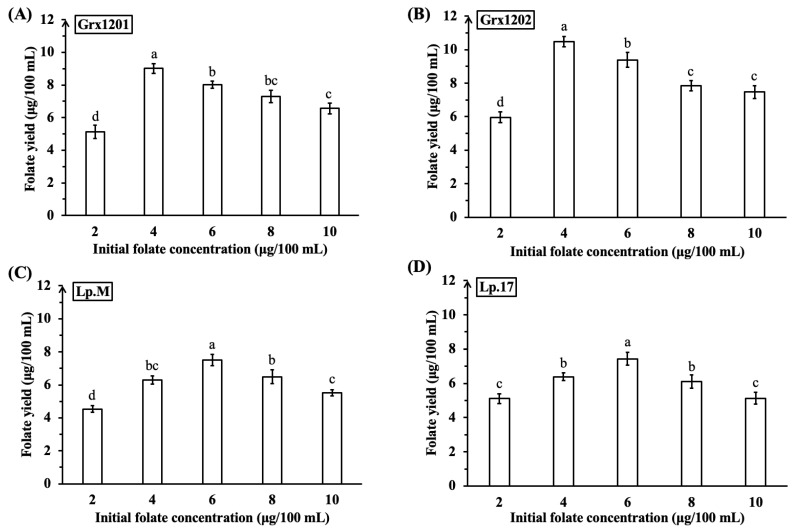
Folate yields under different initial folate concentrations: (**A**) Grx1201, (**B**) Grx1202, (**C**) Lp.M, and (**D**) Lp.17. ^a,b,c,d^ Different small letters denote significant differences (*p* < 0.05).

Folate is not only a metabolite but also acts as a growth factor for lactobacilli [41,43]. Therefore, different concentrations of folate were added to FAAM to determine their effects on the growth and folate synthesis of screened strains. The growth curve of strains Grx1201, Grx1202, Lp.M, and Lp.17 in FAAM was shown in Figure 2A–D. When supplemented with 2, 4, 6, 8, or 10 μg/100 mL of folate, the growth curve had no obvious change, indicating that folate supplementation had no significant effect on growth. As shown in Figure 3A–D, folate production by Grx1201, Grx1202, Lp.M, and Lp.17 first increased and then decreased with increasing folate supplementation, and all values were higher than those of the control, suggesting that folate accumulation occurred. When 4 μg/100 mL of exogenous folate was added to FAAM, Grx1201 and Grx1202 achieved maximum folate yields of 9.01 μg/100 mL and 10.47 μg/100 mL, respectively, which were 2.30- and 2.14-fold higher than those of the control. In contrast, the maximum folate yields of Lp.M and Lp.17 were 7.52 μg/100 mL and 7.43 μg/100 mL, respectively, in FAAM supplemented with 6 μg/100 mL of exogenous folate, which were lower than those of Grx1201 and Grx1202. Compared with Grx1201 and Grx1202, Lp.M and Lp.17 required higher initial folate concentrations to achieve the maximum folate yields, which may increase production costs. Based on these results, Grx1201 and Grx1202 were selected for subsequent experiments. Greppi et al. (2017) observed that *L. fermentum* 8.2 and *L. plantarum* 6.2 achieved the highest folate yields (9.7 μg/100 mL and 9.3 μg/100 mL) in rich folate medium [40], and the folate yield of Grx1202 in the present study was higher under the tested conditions. Passion fruit by-product and fructooligosaccharides could be used as dietary ingredients to promote the folate production [44]. Addition of folate precursors including *p*ABA, glutamate, and GTP to the fermentation medium increased the production of L-5-methyltetrahydrofolate [45]. To further improve the folate production of Grx1201 and Grx1202, passion fruit by-product, fructooligosaccharides, and folate precursors may be supplemented to the medium in future research.

### 3.3. Species Identification of Grx1201 and Grx1202

**Figure 4:**
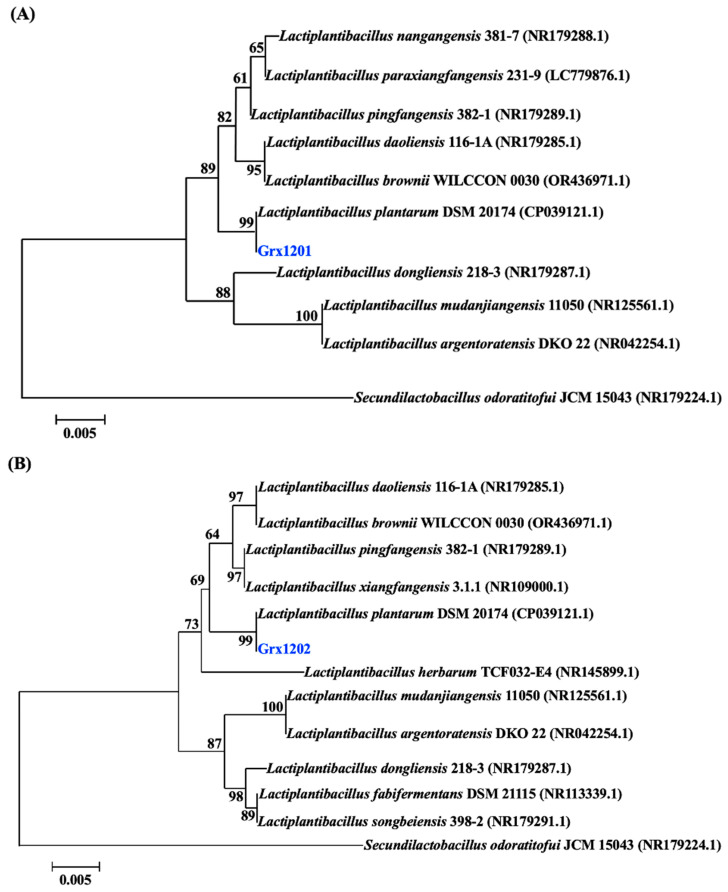
Phylogenetic tree showing the relationships among identified taxa and type strains of each species based on 16S rDNA sequences: (**A**) Grx1201, (**B**) Grx1202. *Secundilactobacillus odoratitofui* JCM 15043 was used as an outgroup. The tree was made using the neighbor-joining method with maximum composite likelihood model in MEGA 10. Only bootstrap values > 60% are indicated. Genbank accession numbers are shown following the organism’s name in parentheses. The scale bar indicates 0.005 nucleotide changes per position.

To determine the species, Grx1201 and Grx1202 were first subjected to the API 50 CHL test to identify lactobacilli based on phenotype characteristics. The carbohydrate fermentation result of Grx1201 and Grx1202 was summarized in Table 2. API software analysis revealed that Grx1201 and Grx1202 belong to *L. plantarum*. Subsequently, the 16S rDNA sequences of Grx1201 and Grx1202 were amplified using specific primers with genomic DNA as a template. Two specific bands of about 1500 bp were observed by agarose gel electrophoresis (Supplementary Figure S1). The 16S rDNA sequences were then analyzed and compared with those in the GenBank database. Homology analysis showed that the 16S rDNA sequences of Grx1201 and Grx1202 shared 98.28% and 100% similarity with *L. plantarum* TMPC 3V122 (Accession number: OM617981.1) and *L. plantarum* 019 (Accession number: JN560843.1). Grx1201 and Grx1202 were identified as *L. plantarum* by China General Microbiological Culture Collection Center (CGMCC) with the CGMCC number of 31048 and 31049. The phylogenetic grouping in Figure 4 further supports the identification of Grx1201 and Grx1202 as *L. plantarum*.

### 3.4. Evaluation of Acid and Bile Salt Resistance of Grx1201 and Grx1202

Acid and bile salt resistance analyses are important for evaluating the survival of lactic acid bacteria in the gastrointestinal tract, providing a basis for functional food development [46]. Therefore, the acid and bile salt resistance of Grx1201 and Grx1202 was evaluated. As shown in Table 3, the survival rates of Grx1201 and Grx1202 were 94.71% and 83.28% in simulated gastric juice at pH 3.0, indicating strong acid resistance under these conditions. When the pH was reduced to 2.0, the survival rates of Grx1201 and Grx1202 decreased to 18.47% and 6.06%, respectively, indicating reduced survival under extremely acidic conditions. Moreover, the survival rates of Grx1201 and Grx1202 were 38.19% and 26.16%, respectively, in medium supplemented with 0.1% (*w*/*v*) bile salt, suggesting moderate bile salt resistance. When bile salt concentration increased to 0.3% (*w*/*v*), survival rates declined to 0.97% and 2.66%, respectively, indicating poor bile salt resistance at high bile salt concentrations. To further improve the survival rate of Grx1201 and Grx1202 under strong acidity and high bile salt concentration environment, acid and bile salt resistant domestication should be considered in the future. Identification of the acid and bile salt resistant genes in Grx1201 and Grx1202 via genomic technique, and then modification of the enzymatic properties of encoded proteins via directed evolution strategy to strengthen the adaptability to acid and bile salt may be another effective way.

### 3.5. Biosafety Evaluation of Grx1201 and Grx1202

#### 3.5.1. Antibiotic Resistance Analysis

Antibiotic resistance in microorganisms may indicate the presence of resistance genes [47]. If resistance genes are transferred to pathogenic bacteria, treatment of infections using commonly applied antibiotics may become less effective, posing potential health risks. Therefore, antibiotic resistance profiling is important for evaluating the biosafety of strains intended for use in fermented foods. Based on this, the antibiotic resistance of Grx1201 and Grx1202 was evaluated. Grx1201 and Grx1202 showed resistance only to vancomycin, penicillin, and ciprofloxacin among the tested antibiotics (Table 4), indicating the relevant resistance genes existed in their genomes. To determine whether Grx1201 and Grx1202 meet international probiotic safety guidelines, clinical trials should be implemented in future study to confirm whether these resistance genes are transferable. For the other nine antibiotics, including tetracycline, gentamicin, chloramphenicol, erythromycin, ampicillin, cefazolin, rifampicin, co-trimoxazole, and clindamycin, sensitivity or intermediate responses were observed, suggesting that resistance determinants were absent or weakly expressed. The food-grade strain *L. rhamnosus* GG showed resistance to four antibiotics, including gentamicin, vancomycin, ampicillin, and co-trimoxazole. These results indicated that Grx1201 and Grx1202 were comparable to *L. rhamnosus* GG in antibiotic resistance safety.

#### 3.5.2. Analysis of the Biogenic Amine-Synthesizing Ability

**Figure 5:**
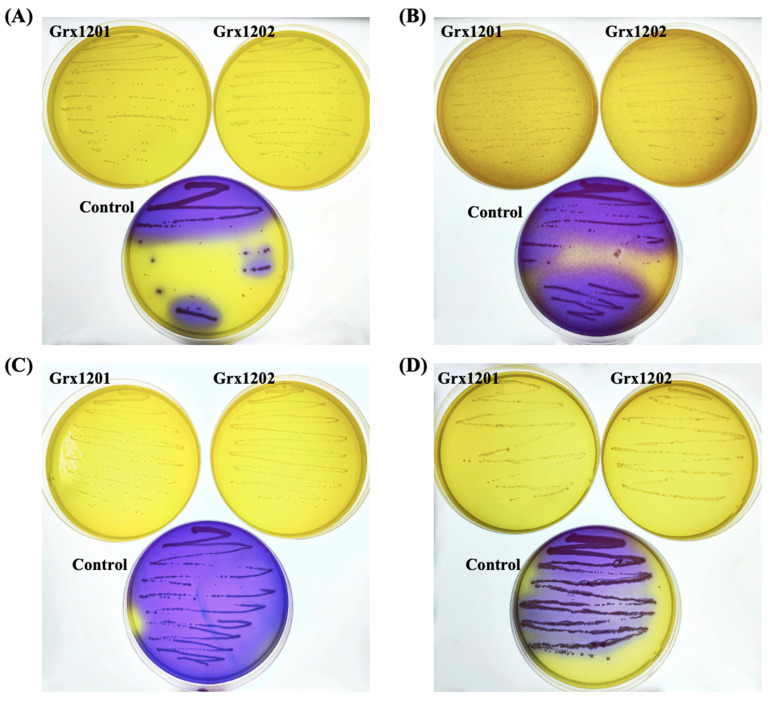
Determination of biogenic amine-synthesizing ability of Grx1201 and Grx1202. Biogenic amine test media supplemented with (**A**) lysine, (**B**) tyrosine, (**C**) ornithine, and (**D**) histidine. Strain *S. aureus* ATCC 6538 was used as a control.

Biogenic amines are nitrogen-containing organic compounds with low molecular weight. Appropriate levels of biogenic amines in foods may support normal physiological functions, whereas high doses may cause adverse symptoms such as migraine and palpitations [48,49]. Therefore, the biogenic amine-synthesizing abilities of Grx1201 and Grx1202 were assessed. As shown in Figure 5A–D, when Grx1201 and Grx1202 were inoculated on biogenic amine test media containing lysine, tyrosine, ornithine, or histidine, the media remained yellow after incubation at 37 °C for 48 h, with no obvious color changes compared with the pre-inoculation state. These results indicated that Grx1201 and Grx1202 did not produce lysine decarboxylase, tyrosine decarboxylase, ornithine decarboxylase, or histidine decarboxylase, and therefore did not synthesize cadaverine, tyramine, putrescine, or histamine. In contrast, *S. aureus* ATCC 6538 used as a control produced purple colonies on the test media, indicating the production of decarboxylases involved in biogenic amine formation. Overall, these results indicated that Grx1201 and Grx1202 did not synthesize biogenic amines. Zeng et al. (2023) found that *L. plantarum* strains ZL-3, ZL-14, ZL-44 and *L. pentosus* strain ZL-22 grown on biogenic amine assay medium supplemented with 0.5% (*w*/*v*) histidine, 0.5% (*w*/*v*) lysine, 0.04% (*w*/*v*) tyrosine, and 0.5% (*w*/*v*) ornithine produced 7.339 × 10^3^ to 9.405 × 10^3^ μg/100 mL of biogenic amines after cultured at 30 °C for 96 h [50]. Compared with this report, Grx1201 and Grx1202 have better biosafety.

#### 3.5.3. Hemolytic Ability Analysis

**Figure 6:**
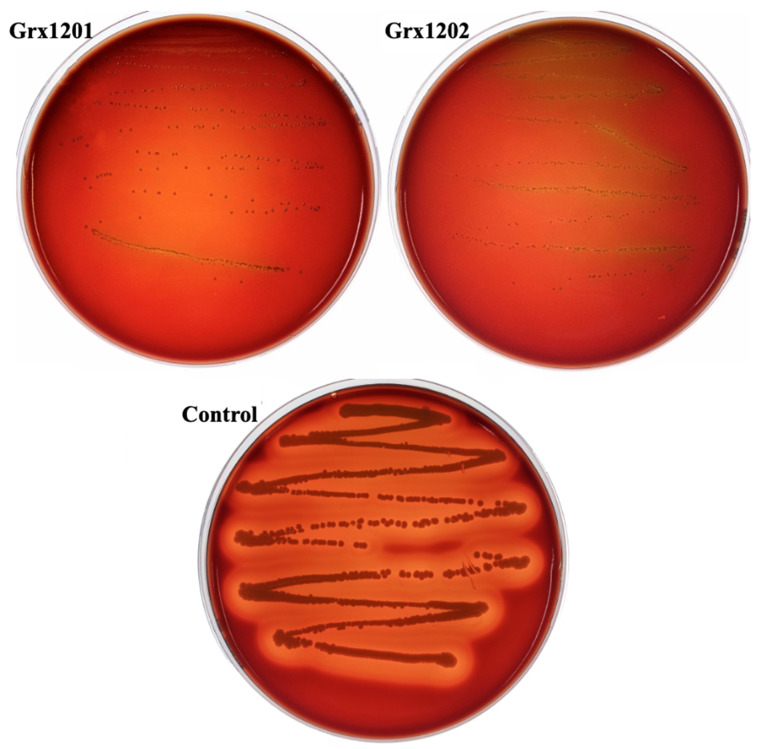
Hemolytic ability analysis of Grx1201 and Grx1202. Strain *S. aureus* ATCC 6538 was used as a control.

Microorganisms with hemolytic ability can produce hemolysins that disrupt animal blood cells, leading to hemolysis [51]. For strains intended for food applications, hemolytic activity is an important biosafety indicator. Therefore, the hemolytic activities of Grx1201 and Grx1202 were examined. As shown in Figure 6, no transparent zones were observed around colonies of Grx1201 and Grx1202 on Columbia agar containing sheep blood after incubation at 37 °C for 48 h, indicating the absence of hemolytic activity. In contrast, *S. aureus* ATCC 6538 used as a positive control produced transparent zones around colonies, confirming its hemolytic capability. These results indicated that Grx1201 and Grx1202 exhibited no hemolytic activity. The *L. plantarum* AHQ-14 isolated from Chinese Xinjiang district traditional dairy products and *L. bulgaricus* BD0390 donated by Bright Dairy Company (Shanghai, China) were gamma-hemolytic [52]. Compared with the reported strains, Grx1201 and Grx1202 are more suitable for biological applications.

### 3.6. Analysis of Key Genes in the Folate Synthesis Pathway

**Figure 7:**
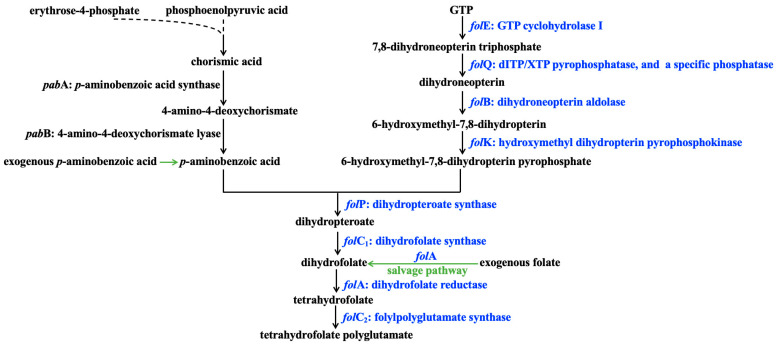
Folate synthesis pathway in lactic acid bacteria. Key genes and their encoded proteins in the pterin branch were marked as blue.

The folate synthesis pathway in lactic acid bacteria consists of *p*ABA branches and pterin branches (Figure 7), and folate synthesis requires the coordinated function of both branches. *p*ABA is an important precursor for folate synthesis. Although most lactic acid bacteria lack a complete de novo pathway for *p*ABA synthesis, *p*ABA can be assimilated from FAAM through a salvage route [53,54]. Since *p*ABA is available from the medium, the pterin branch is considered to contribute primarily to de novo folate synthesis. To explore the basis of high folate production in Grx1201 and Grx1202, key genes in the pterin branch were identified, and their transcription levels were analyzed.

**Figure 8:**
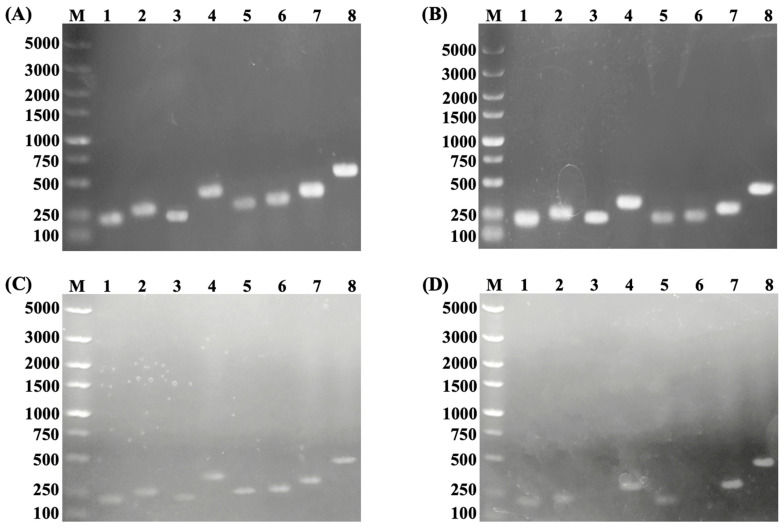
Agarose gel electrophoresis of key genes in the pterin branch. Key genes derived from (**A**) Grx1201, (**B**) Grx1202, (**C**) Lp.621, and (**D**) Lp.P9. M: marker; 1: *fol*Q; 2: *fol*B; 3: *fol*K; 4: *fol*A; 5: *fol*P; 6: *fol*E; 7: *fol*C_1_; 8: *fol*C_2_.

As shown in Figure 8A,B, agarose gel electrophoresis indicated that the amplified products of *fol*E, *fol*Q, *fol*B, *fol*K, *fol*P, *fol*C_1_, *fol*A, and *fol*C_2_ matched the reported theoretical sizes [54]. These genes encode GTP cyclohydrolase I, dITP/XTP pyrophosphatase, dihydroneopterin aldolase, hydroxymethyl dihydropterin pyrophosphokinase, dihydropteroate synthase, dihydrofolate synthase, dihydrofolate reductase, and folylpolyglutamate synthase, respectively, indicating their presence in the genomes of Grx1201 and Grx1202. Therefore, Grx1201 and Grx1202 can synthesize folate de novo through the pterin branch. When low-folate-yielding strains Lp.621 and Lp.P9 were used as controls (Figure 8C,D), specific bands corresponding to all eight key genes were detected in Lp.621. In contrast, *fol*K and *fol*E were not detected in Lp.P9. However, folate production was still observed in Lp.P9, suggesting the possible presence of isozyme genes with functions similar to those of *fol*K and *fol*E.

**Figure 9:**
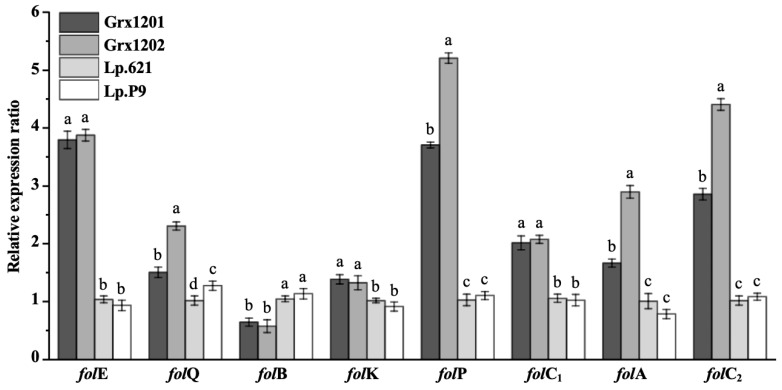
Analysis of the transcription levels of key genes in the pterin branch derived from Grx1201, Grx1202, Lp.621, and Lp.P9. ^a,b,c,d^ Different small letters denote significant differences (*p* < 0.05).

The transcription levels of key genes in the pterin branch were assessed by qRT-PCR. As shown in Figure 9, except for *fol*B, the transcription levels of *fol*E, *fol*Q, *fol*K, *fol*P, *fol*C_1_, *fol*A, and *fol*C_2,_ in Grx1201 and Grx1202 were higher than those in Lp.621 and Lp.P9, which may increase the synthesis of enzymes involved in the pterin branch and promote folate formation from GTP. The transcription levels of *fol*E, *fol*B, *fol*K, and *fol*C_1_ were comparable between Grx1201 and Grx1202. In contrast, the transcription levels of *fol*Q, *fol*P, *fol*A, and *fol*C_2_ in Grx1202 were 1.53-, 1.40-, 1.74-, and 1.54-fold higher than those in Grx1201, which may enhance the abundance of the corresponding proteins and facilitate de novo folate synthesis. Dihydrofolate reductase encoded by *fol*A catalyzes the conversion of dihydrofolate to tetrahydrofolate and may also contribute to the conversion of exogenous folate to dihydrofolate through a salvage pathway [55]. Under identical supplementation conditions, the higher folate yield in Grx1202 compared with Grx1201 may be associated with elevated *fol*A transcription. The extracellular folate yield of the dairy starter bacterium *L. lactis* MG1363 increased almost 10-fold through *fol*E overexpression [56]. In addition, folic acid production by the industrial fungus *Ashbya gossypii* increased 16-fold through simultaneous overexpression of *AgFOL1*, *AgFOL2*, and *AgFOL3* [14]. The dihydrofolate yield increased through inhibition of the activity of dihydrofolate reductase from HMA07 strain [57]. These findings suggest that overexpression of *fol* genes or modulation of the catalytic activities of enzymes encoded by *fol* genes may be a feasible strategy to further enhance folate production by Grx1201 and Grx1202 in future studies.

## 4. Conclusions

We screened two folate-producing *L. plantarum* bacteria, Grx1201 and Grx1202, with FAAM. When 4 μg/100 mL of exogenous folate was added to FAAM, the maximum folate production by Grx1201 and Grx1202 reached 9.01 μg/100 mL and 10.47 μg/100 mL, respectively, which were 2.30 and 2.14 times greater than those of the control. High survival rates of Grx1201 (94.71%) and Grx1202 (83.28%) were observed at pH 3.0, indicating that they have strong acid resistance. When cultured in medium supplemented with 0.1% (*w*/*v*) bile salt, the survival rates of Grx1201 and Grx1202 reached 38.19% and 26.16%, respectively, indicating that they have moderate bile salt resistance. In terms of antibiotic resistance, biogenic amine synthesis, and hemolytic ability, Grx1201 and Grx1202 were found to be safe for biological application. Higher transcription of the target genes *fol*E, *fol*Q, *fol*K, *fol*P, *fol*C_1_, *fol*A, and *fol*C_2_ in Grx1201 and Grx1202 facilitates the metabolic synthesis of folate from GTP. The strains Grx1201 and Grx1202 reported here have great potential for improving the folate content in functional foods.

## Figures and Tables

**Table 1:** The extracellular folate yields of 18 isolates with OD_580_ > 1.0 after 16 h of growth in FAAM.

Strains	Folate Yield (μg/100 mL)	Strains	Folate Yield (μg/100 mL)
Lp.g	ND	Lp.X2	ND
Lp.58	ND	Lp.4	ND
Lp.11	ND	Lp.7	ND
Lp.d	ND	Lp.154	ND
Lp.10	ND	Lp.a	ND
Grx1201	3.91 ± 0.45 ^ab^	Lp.621	1.64 ± 0.21 ^c^
Lp.M	3.42 ± 0.40 ^b^	Lp.P9	1.58 ± 0.22 ^c^
Lp.113	ND	Grx1202	4.89 ± 0.27 ^a^
Lp.54	ND	Lp.17	3.55 ± 0.39 ^b^

Note: ND = Not detected. ^a,b,c^ Different small letters denote significant differences (*p* < 0.05).

**Table 2:** Identification of Grx1201 and Grx1202 using API 50 CHL kit.

No	Substrates	Grx1201	Grx1202	No	Substrates	Grx1201	Grx1202
0	Control	−	−	25	Esculin	+	+
1	Glycerol	−	−	26	Salicin	+	+
2	Erythritol	−	−	27	D-Cellobiose	+	+
3	D-arabinose	−	−	28	D-Maltose	+	+
4	L-arabinose	+	+	29	D-Lactose	+	+
5	Ribose	−	+	30	D-Melibiose	+	+
6	D-xylose	−	+	31	Sucrose	+	+
7	L-xylose	−	−	32	Trehalose	+	+
8	D-Adonitel	−	−	33	Inulin	+	−
9	Bmethyl-D-Xyloside	−	−	34	Melezitose	+	+
10	D-Galactose	+	+	35	Raffinose	+	−
11	D-Glucose	+	+	36	Starch	−	−
12	D-Fructose	+	+	37	Glycogen	−	−
13	Mannose	+	+	38	Xylitol	−	−
14	Sorbose	−	−	39	B-Gentiobiose	+	+
15	L-Rhamnose	+	+	40	D-turanose	+	+
16	Dulcitol	−	−	41	D-Lyxose	−	−
17	Inositol	−	−	42	D-tagatose	−	−
18	Mannitol	+	+	43	D-fucose	−	−
19	Sorbitol	+	+	44	L-fucose	−	−
20	Methyl-D-mannoside	+	+	45	D-arabitol	−	−
21	Metyl-D-glucoside	−	−	46	L-arabitol	−	−
22	N-Acetyl-Glucosamine	+	+	47	Gluconate	+	+
23	Amygdalin	+	+	48	2-Keto-Gluconate	−	−
24	Arbutin	+	+	49	5-Keto-Gluconate	−	−

+: positive, −: negative.

**Table 3:** Analysis of the acid and bile salt resistance of Grx1201 and Grx1202.

Strains	Survival Rate (%)			
	Acidity of Simulated Gastric Juice		Bile Salt Concentration	
	pH 2.0	pH 3.0	0.1% (*w*/*v*)	0.3% (*w*/*v*)
Grx1201	18.47 ± 0.56 ^a^	94.71 ± 0.73 ^a^	38.19 ± 1.33 ^a^	0.97 ± 0.13 ^b^
Grx1202	6.06 ± 0.26 ^b^	83.28 ± 1.43 ^b^	26.16 ± 0.73 ^b^	2.66 ± 0.22 ^a^

Note: ^a,b^ Different small letters denote significant differences (*p* < 0.05).

**Table 4:** Antibiotic resistance analysis of Grx1201 and Grx1202. Food-grade strain *L. rhamnosus* GG was used as a control.

Antibiotics	Resistance			Antibiotics	Resistance		
	Grx1201	Grx1202	*L. rhamnosus* GG		Grx1201	Grx1202	*L. rhamnosus* GG
Tetracycline	S	I	S	Penicillin	R	R	S
Gentamycin	I	S	R	Cefazolin	S	S	S
Chloramphenicol	S	S	S	Ciprofloxacin	R	R	I
Erythromycin	S	I	S	Rifampicin	I	S	S
Vancomycin	R	R	R	Co-trimoxazole	S	S	R
Ampicillin	S	S	R	Clindamycin	S	I	S

Note: S, I, and R represent sensitivity, intermediate, and resistance, respectively.

## Data Availability

The original contributions presented in this study are included in the article/Supplementary Material. Further inquiries can be directed to the corresponding author.

## Supplementary Materials

The following supporting information can be downloaded at: https://www.mdpi.com/article/10.3390/foods15101705/s1. Table S1: Strains used in this study. Table S2: Primers used in this study. Table S3: Antibiotic content in the tablet and resistance assessment criteria. Table S4: Components of the biogenic amine test medium. Figure S1: Agarose gel electrophoresis of 16S rDNAs from Grx1201 and Grx1202. M: marker; 1: Grx1201; 2: Grx1202.
