# Supplementary material for: Screening, Identification, and Characterization of Two Folate-Producing Lactiplantibacillus plantarum Strains
Source: Foods. 2026 May 13;15(10):1705. doi: 10.3390/foods15101705 (PMC13205532; doi:10.3390/foods15101705)
Supplement: Supplementary file 1 [file foods-15-01705-s001.zip › foods-4255404-supplementary.pdf]

Screening, identification, and characterization of two folate-producing *Lactiplantibacillus plantarum* strains

Bo Pang [1,2], Haobin Mo [1,2], Wenxin Zhang [1,2], Wenqiong Wang [1,2], Dawei Chen [1,2], Ruixia Gu [1,2], and Yujun Huang [1,2*]

[1] School of Food Science and Engineering, Yangzhou University, Yangzhou 225127, Jiangsu, China

[2] Key Laboratory of Probiotics and Deep Processing of Dairy Products, Yangzhou University, Yangzhou 225127, Jiangsu, China

*Corresponding author: Yujun Huang

E-mail address: yjhuang@yzu.edu.cn

Tel. & fax: +86-514-87978128

Table S1. Strains used in this study.

Strains	Sources	Strains	Sources
Lp.67	Human feces	Lp.B28	Milk fan
Lp.g	Pickle	Lp.X2	Chimichurri sauce
Lp.58	Human feces	Lp.39	Human feces
Lp.11	Human feces	Lp.4	Human feces
Lp.h	Chimichurri sauce	Lp.7	Marinated cucumber
Lp.d	Pickle	Lp.154	Human feces
Lp.10	Pickle	Lp.a	Chimichurri sauce
Grx1201	Pickle	Lp.621	Pickle
Lp.M	Pickle	Lp.117	Human feces
Lp.113	Human feces	Lp.P9	Pickle
Lp.c	Marinated cucumber	Grx1202	Pickle
Lp.H5	Marinated cucumber	Lp.17	Marinated cucumber
Lp.54	Human feces	Lp.667	Pickle

Table S2. Primers used in this study.

Primers	Sequences (5'→3')
16-F	AGAGTTTGATCCTGGCTCAG
16-R	GGCTGCTGGCACGTAGTTAG
*fol*E-F	CGGGTTGCACGAATGTATGC
*fol*E-R	ACTGTCAACCGCTCCTGAAC
*fol*Q-F	GGCTTGACTGCTCGTCAGTA
*fol*Q-R	TGACTGCAACCCCTAAGTCG
*fol*B-F	GGAAGAACGGCGTAATGGTC
*fol*B-R	TTCCAGGCATTGGTACGCTA
*fol*K-F	CCATTTCCAGGTGGGGAATC
*fol*K-R	GGGGTGGTCCAAGCAAACTT
*fol*P-F	CCASGRCSGCTTGCATGAC
*fol*P-R	TKACGCCGGACTCCTTTTWY
folC_1-F	AGTGAGCGATTTGGACAGCA
folC_1-R	AGTCGCTGCCATCCTTGAAA
*fol*A-F	GACATGCAGCGGTTCAAAGC
*fol*A-R	ACCGTCCCAATTTGTTGGCT
folC_2-F	GGCTGTTTTGCAGACCGAAG
folC_2-R	TGCGGGCGTATTCGTAATCA

The degenerate bases S, R, K, W, and Y represent C or G, A or G, T or G, A or T, and C

or T, respectively.

Table S3. Antibiotic content in the tablet and resistance assessment criteria.

Antibiotics	Content (μg per tablet)	Diameter of inhibition zone (mm)		
		S	I	R
Tetracycline	30	19	15–18	14
Gentamycin	10	15	12–14	11
Chloramphenicol	30	18	13–17	12
Clindamycin	15	19	16–18	15
Erythromycin	15	23	14–22	13
Vancomycin	30	17	15–16	14
Ampicillin	10	29	-	28
Penicillin	10	29	-	28
Cefazolin	30	18	15–17	14
Ciprofloxacin	5	21	16–20	15
Rifampicin	5	20	17–19	16
Co-trimoxazole	23.75	16	11–15	10

Note: S, I, and R represent sensitivity, intermediate, and resistance, respectively.

Table S4. Components of the biogenic amine test medium.

Reagents	Concentration (μg/100 mL)	Reagents	Concentration (μg/100 mL)
Tryptone	5×10^5	$FeSO_4$	4×10^3
Yeast extract	5×10^5	K_2HPO_4	2×10^5
Beef extract	5×10^5	Vitamin B1	10^3
NaCl	2.5×10^5	$CaCO_3$	10^4
Glucose	5×10^4	Pyridoxal phosphate	5×10^3
Tween-80	10^5	Bromocresol violet	6×10^3
$MgSO_4$	2×10^4	Agar powder	2×10^6
$MnSO_4$	5×10^3		

Figure S1

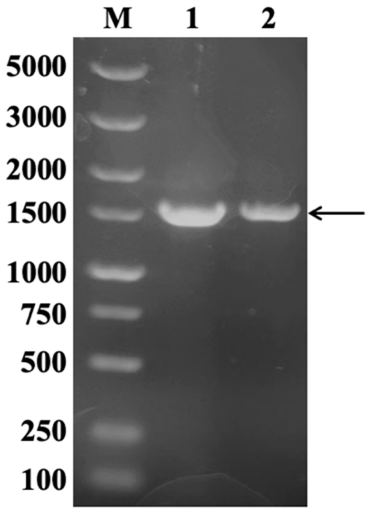

Figure S1. Agarose gel electrophoresis of 16S rDNAs from Grx1201 and Grx1202. M: marker; 1: Grx1201; 2: Grx1202.
